# The Transcriptional Response of *Drosophila melanogaster* to Infection with the Sigma Virus (Rhabdoviridae)

**DOI:** 10.1371/journal.pone.0006838

**Published:** 2009-08-31

**Authors:** Jennifer Carpenter, Stephan Hutter, John F. Baines, Julia Roller, Sarah S. Saminadin-Peter, John Parsch, Francis M. Jiggins

**Affiliations:** 1 Institute of Evolutionary Biology, University of Edinburgh, Edinburgh, United Kingdom; 2 Section of Evolutionary Biology, Department of Biology, University of Munich, Planegg-Martinsried, Germany; Charité-Universitätsmedizin Berlin, Germany

## Abstract

**Background:**

Bacterial and fungal infections induce a potent immune response in *Drosophila melanogaster*, but it is unclear whether viral infections induce an antiviral immune response. Using microarrays, we examined the changes in gene expression in Drosophila that occur in response to infection with the sigma virus, a negative-stranded RNA virus (Rhabdoviridae) that occurs in wild populations of *D. melanogaster*.

**Principal Findings:**

We detected many changes in gene expression in infected flies, but found no evidence for the activation of the Toll, IMD or Jak-STAT pathways, which control immune responses against bacteria and fungi. We identified a number of functional categories of genes, including serine proteases, ribosomal proteins and chorion proteins that were overrepresented among the differentially expressed genes. We also found that the sigma virus alters the expression of many more genes in males than in females.

**Conclusions:**

These data suggest that either Drosophila do not mount an immune response against the sigma virus, or that the immune response is not controlled by known immune pathways. If the latter is true, the genes that we identified as differentially expressed after infection are promising candidates for controlling the host's response to the sigma virus.

## Introduction

Viral infections in arthropods are widespread and are of considerable economic and medical importance. For example, viruses have had devastating economic consequences on honey-bee and shrimp populations [1], [2], and many viral pathogens in humans, crops and livestock are vectored by insects. Carefully chosen model systems could provide great insight into how arthropods combat viral infections. The principal model organism used to study invertebrate immune defenses is the fruit fly *Drosophila melanogaster* [3]. Although many studies have examined Drosophila's defenses against bacterial and fungal infections, relatively little is known about antiviral defenses. This is despite *D. melanogaster* being infected with a diverse range of viruses, including positive-stranded RNA viruses (several picornaviruses, including the Drosophila C virus), a negative-stranded RNA virus (sigma virus; Rhabdoviridae), and a double-stranded RNA virus (DFV; Reoviridae) [4].

The viruses that infect *Drosophila* have very different lifecycles and biology, which may have important implications for immune recognition and the immune response. The picornaviruses are released by lysing host cells, and the viral particles are non-enveloped [5]. In contrast, the Rhabdovirus sigma is released from host cells by budding, and the viral particles are enclosed in a lipid envelope with surface-exposed glycoproteins [4], [6]. Furthermore, the picornavirus DCV can cause severe pathology in infected flies, while the sigma virus is relatively benign [4]. Antiviral immune responses often recognize RNA viruses by the presence of dsRNA. Typically, positive-sense RNA viruses produce much more double stranded RNA than negative sense viruses, probably because the nucleocapsid protein of negatively sensed RNA viruses can prevent the two strands from annealing to produce dsRNA [7]. All of these factors may mean that the mechanisms by which flies can recognize viruses and protect themselves against infection may be differ between different viruses.

The only immune effector that has been found to target viruses in Drosophila is RNAi [8]–[10]. RNAi can distinguish self from non-self because RNA viruses often produce double-stranded RNA, while host cells typically do not. Some RNA viruses have a double-stranded genome, and even single stranded RNA viruses can produce double-stranded RNA during replication, gene expression or as a consequence of RNA secondary structure. RNAi processes this double-stranded RNA into short fragments, which are passed to an effector complex that recognizes and degrades viral RNA with the complimentary sequence. RNAi pathway genes are constitutively expressed, and are not known to be up-regulated by viral infection [11].

Other studies have examined the changes in gene expression that occur when flies are infected by viruses [11]–[13]. Microarray analyses of flies injected with DCV identified many up-regulated genes, raising the possibility that there is an induced immune response to viruses in addition to RNAi [11]. Detailed studies of one of the up-regulated genes identified in this study, *vir-1*, revealed that it was under the control of the Jak-STAT pathway, which is an important component of the antiviral response of vertebrates. Not only does DCV activate this pathway, but flies that are deficient for the Jak-kinase Hopscotch have a higher viral load and lower survival than wild-type flies [11]. It is currently unknown how the Jak-STAT pathway detects viral infection, or how it protects flies against DCV (knocking down *vir-1* expression does not make flies more susceptible to DCV).

In addition to the Jak-STAT pathway, the Toll pathway, an immune signaling pathway activated by bacteria and fungi, has been implicated in antiviral immunity. A previous study has shown that the Drosophila X virus (DXV) activates the Toll pathway, and flies that are deficient for the Toll pathway transcription factor *Dif* are more susceptibility to DXV infection [13]. Furthermore, a gene called *ref(2)P*, which is required by the Toll immune response, has a naturally occurring polymorphism that reduces the rate at which sigma virus replicates within the fly [14], [15]. However, the role of the Toll pathway as a antiviral response remains uncertain because previous studies have shown that Toll pathway genes are not up-regulated by DCV infection [11], and not all Toll pathway mutants alter the flies' susceptibility to DXV [13].

In this study we have used microarrays to see which immune pathways, if any, are up-regulated when Drosophila is infected with the sigma virus—a naturally occurring pathogen that infects about 4% of *D. melanogaster* in the wild [16]. The sigma virus is transmitted only vertically, from parent to offspring through both eggs and sperm [4]. Typical for a vertically transmitted pathogen, the sigma virus is a fairly benign infection that causes a slight reduction in the survival and fecundity of infected flies [17]. There is however a great deal of genetic variation in the susceptibility of flies to sigma infection in natural populations [18], [19]. Several major effect resistance genes have been mapped [4], but only one of these has been identified (*ref(2)P*; see above). It is currently unclear whether these resistance genes are part of an antiviral immune response mounted by the flies or are host molecules that the virus exploits for its own replication and transmission. The only study which looked at the transcriptional response to sigma virus infection measured the expression of 15 immunity-related genes using quantitative real-time PCR (qPCR), and found that several antimicrobial peptides and the peptidoglycan recognition proteins (PGRP-SB1 and PGRP-SD) are up-regulated in infected flies [20]. However, it is unclear whether this transcriptional response has any effect on the replication or transmission of the virus.

## Materials and Methods

### Drosophila stocks and hybridizations

We compared patterns of gene expression in genetically identical flies that were either infected or uninfected with the sigma virus. The flies were an isogenic stock *SM5/Pm*;*spa^pol^* that had been infected with the sigma virus isolate AP30 several generations before (see [19] for details). These flies have the susceptible allele of the *ref(2)P* gene which controls sigma virus replication. Four replicates of both the infected and uninfected flies were reared on Lewis medium [21] at a constant density of approximately 220 flies per bottle at 25°C on a 12 hour light-dark cycle for a minimum of three generations before the experiment. The flies were aged and allowed to mate for six days before being sexed on ice (they were not exposed to CO_2_), and RNA was extracted from pools of 180 males or 60 females using Trizol (Invitrogen, Carlsbad, CA, USA). We confirmed the infection status of a sample of flies from the same bottles using a standard CO_2_ test [19].

We performed 5 dye swap replicates (10 arrays) on the males and 4 dye swap replicates (8 arrays) on the females. Each dye-swap compared a different pair of RNA extractions from sigma-infected and -uninfected flies (*i.e.*, biological replicates). A more detailed description of the hybridizations and statistical analysis is given by Hutter *et al.* [22], who used many of the same methods. We used a *D. melanogaster* microarray obtained from the Drosophila Genomics Resource Center (DGRC; Bloomington, IN, USA) known as DGRC-1. This consists of 13,921 exonic PCR amplicons (100–600 bp in length) representing 11,895 unique genes (∼88% of the genome).

The RNA was reverse transcribed and labeled with the SuperScript Plus Indirect cDNA Labeling System and Alexa Fluor 555 and 647 dyes (Invitrogen, Carlsbad, CA, USA). Hybridizations were performed following DGRC protocols and arrays were scanned using an aQuire microarray scanner (Genetix, New Milton, UK). All array data have been submitted to the Gene Expression Omnibus database (http://www.ncbi.nlm.nih.gov/geo) under series XXX.

### Data analysis

To correct each spot on our arrays for local background effects, within-array variation and between-array variation, we normalized the signal intensity of the two dye channels using the three-step procedure described by [22] and implemented in CARMAweb [23]. In short, the relative expression level and probability of differential expression between infected and uninfected flies for each gene was estimated using the Markov Chain Monte Carlo algorithm implemented in BAGEL [24]. The results of the BAGEL runs are provided as Supporting Files S1 and S2. As some genes are represented by multiple probes, we defined a gene as differentially expressed if at least one of its probes displayed a significant difference. For each slide, only those spots displaying a signal greater than 95% of the negative control probes (182 probes from other species) in each dye channel were considered ‘expressed’, and ‘non-expressed’ data points were excluded from the analysis. To determine the experiment-wide false discovery rate (FDR), we repeated the BAGEL analysis on a randomized version of our final data-set [22]. To estimate the power of our experiment to detect expression differences between infected and uninfected flies, we calculated the GEL_50_ statistic [25].

To test if any gene ontology (GO) categories were over-represented in our list of differentially expressed genes, we used the web-based tool g:Profiler [26] that corrects for multiple testing while taking the hierarchical nature of GO terms into account. The analysis of gene ontology was based on the annotation of the *D. melanogaster* genome included in release v49 of ENSEMBL [27]. CG numbers were updated to match this version and these lists are provided separately in the Supporting File S3.

### Quantitative real-time PCR

To confirm the results of the microarray analyses, we measured the expression of several genes using qPCR. These genes included *PGRP-SC2* and *Tudor-SN*, whose expression differed between sigma-infected and -uninfected flies in our microarray experiment, and *Attacin-A* and *Drosocin*, which were found to be up-regulated in sigma-infected by Tsai *et al.* [20], but not in our experiments. As an endogenous control, we also measured the expression of *RpL32* (*Rp49*).

To check the infection status of our flies and estimate their viral loads we also amplified viral genomic RNA by qPCR. The primers were designed to amplify a fragment spanning the sigma *N* and *P* genes to ensure that they amplified genomic RNA rather than mRNA. To allow our data on data to be compared to the results of Tsai *et al.* [20], we used *Act88F* as an endogenous control.

For the qPCR, 1.1 µg of total RNA was reverse transcribed using Superscript II reverse transcriptase (Invitrogen) and random hexamer primers. The resulting cDNA was used at 1∶10 dilution for qPCR using TaqMan probes and a 7500 Fast Real-Time PCR System (Applied Biosciences, Foster City, CA, USA). Pre-designed probe IDs were as follows: *PGRP-SC2*: Dm01818611_s1, *Tudor-SN*: Dm01834411_g1, *Attacin-A*: Dm02362218_s1, *Drosocin*: Dm01821449_s1, *RpL32*: Dm02151827_g1 and *Act88F*: Dm02362815_s1. Probes for quantifying viral loads were designed using the Custom TaqMan Assay Design Tool provided by Applied Biosciences. The region included in this assay corresponds to positions 3127 to 3239 of the AP30 isolate sequence (EMBL Accession AM689309) with the following primer and probe sequences: 5′-GCTCACAGTGAAGATCCATTACATG-3′ (forward), 5′- GCGGCTTCACAGAGAATTTGTC-3′ (reverse) and 5′- ACGAGATCTTAGTCAGCACCCT-3′ (probe). Seven replicate assays were performed for each of the 4 treatments: male, female, infected and uninfected and the threshold cycle value (Ct) was averaged across these replicates.

## Results

### Data quality and identification of differentially expressed genes

In total we performed 10 microarray hybridizations on males and 8 on females.

After removing probes that had no signal in either the sigma-infected or -uninfected flies, we were left with 4301 probes representing 3923 genes in females, and 5532 probes representing 4996 genes in males. The complete list of these genes is given in Supporting File S1. The higher number of genes detected as expressed in males may be explained by sex-related differences in gene expression, as we detected expression of a higher proportion of sex-biased genes in males than in females (see Discussion).

To assess the statistical power of the experiment, we calculated the GEL_50_ statistic, which is the fold-change in gene expression at which we have a 50% chance of detecting a difference (*P*<0.05) between the infected and uninfected flies. The GEL_50_ was 1.37 for the males and 1.52 for the females, which is similar to other microarray studies [22].

**Figure 1:**
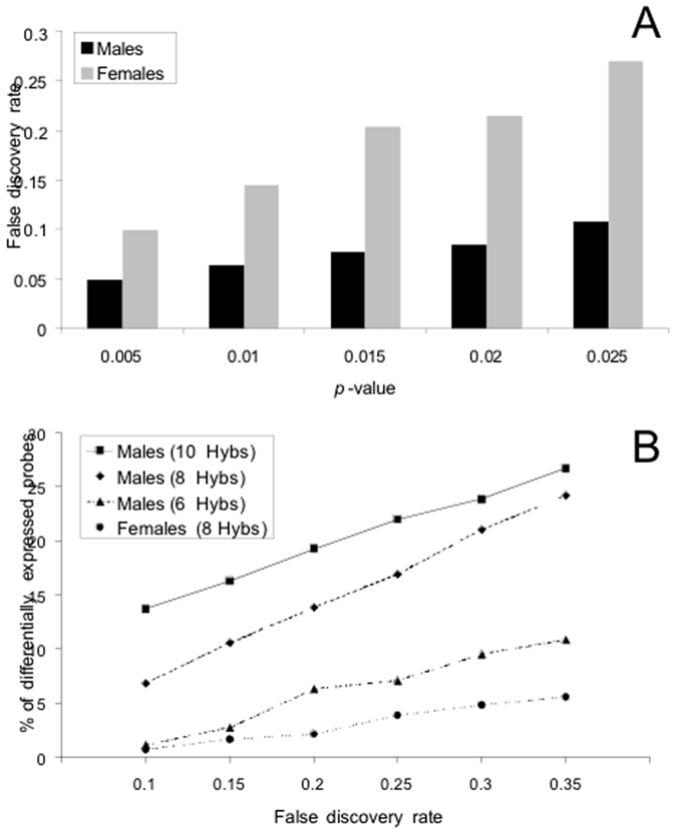
Comparative statistical analysis of the male and female experiments. (A) False discovery rates corresponding to several *P*-value cut-offs for both experiments. (B) Proportion of probes detected as differentially expressed at different false discovery rates. For the male experiment hybridizations which showed the best quality (defined as the number of spots with significant signal above background) were successively removed in order to determine the effect of replication on the detection of expression differences.

In order to define a significance threshold we calculated the FDRs for both the male and female datasets at different *P*-values (Figure 1a). We used a cut-off of *P*<0.02 for the male dataset, which corresponds to an FDR of 8.6%. Using this threshold we found that a total of 629 genes showed expression differences between sigma-infected and –uninfected flies (in infected flies 293 genes were up-regulated and 336 genes were down-regulated). Adjusting the *P*-value to produce a comparable FDR for the female dataset resulted in a very short list of only ∼30 differentially expressed genes. For further analysis we therefore decided to also use a cut-off of *P*<0.02 for females. This corresponds to an FDR of 21% (Figure 1a). At this threshold, we detected 134 differentially expressed genes (in infected flies 46 genes were up-regulated and 88 genes down-regulated). The excess of down-regulated genes in females was statistically significant (χ^2^-test, *P* = 0.0003).

**Figure 2:**
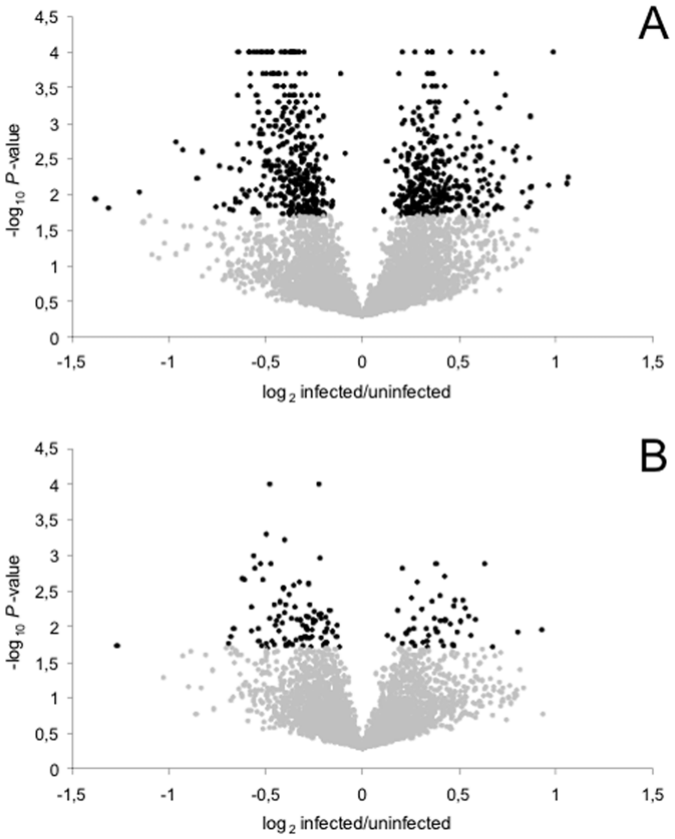
Volcano plots of the (A) male and (B) female experiments. The X-axis defines the magnitude of expression difference between the infected and uninfected state, the Y-axis the corresponding *P*-value of the BAGEL analysis. Probes for which BAGEL assigned a *P*-value of 0 (*i.e.*, *P*<0.0001), were set to *P* = 0.0001. Black dots represent probes up-regulated (log_2_ infected/uninfected>0) or down-regulated (log_2_ infected/uninfected<0) in the infected state at *P*<0.02.

We found that the transcriptional response to sigma infection was very different between males and females. Of the 2862 genes detected as expressed in both males and females, only 41 showed a significant difference in expression in both sexes, and of these, 35 showed a consistent pattern of up- or down-regulation in both sexes (Table 1). Overall the magnitude of these differences was small; for both males and females, the maximum difference in expression was 2.5-fold (Figure 2) and the median difference was 1.28. Despite this, there seems to be a striking difference between males and females in the number of genes affected by infection. In order to investigate if this was due to differences between our replicates we removed the replicate hybridizations with the best quality signal from the male dataset and repeated the calculations. Removing the four best-quality hybridizations lowered the detection sensitivity to below that of the female dataset, and yet, males still showed consistently greater differences in expression (Figure 1b, see Discussion).

**Table 1:** Genes that are either up-regulated or down-regulated in both male and female flies infected with the sigma virus.

Change in expression	Gene name
down	CG9350
down	Ribosomal protein LP1
down	CG6020 (NADH dehydrogenase)
down	CG2875
down	CG1648
down	Serine protease 6
down	CG18594
down	Defensin
down	CG12231
down	CG8311 (dolichol kinase)
down	Ribosomal protein L13A
down	CG7675
down	CG9572
down	Antigen 5-related
down	tre oncogene-related protein
down	CG18179 (serine protease)
down	fau
down	CG4000
down	CG1304 (serine protease)
down	Ard1
down	CG9140
down	yippee interacting protein 7
down	CG7470 (glutamate 5-kinase)
down	PGRP-SC2
down	CG10472 (serine protease)
down	Cytochrome P450-6a2
down	CG17108 (acetyl-CoA carboxylase)
down	CG3088 (serine protease)
down	CG12736 (GTPase)
down	CG12057
down	CG11314
down	CG8343 (mannose binding)
up	rotund
up	Decondensation factor 31
up	Actin 5C

The molecular function of unnamed genes is given in brackets.

### Expression of known immunity genes

Previous studies have shown that a large number of genes are induced when flies are infected with bacteria or fungi, and that many of these genes are under the control of the Toll and IMD immune signaling pathways. To investigate whether these pathways might be activated in response to sigma virus infection, we have compared our results to previous studies that examined the fly's immune response to different pathogens. First, we compared data from a previous microarray study, which examined gene expression in flies infected with bacteria and fungi and identified some 400 up- or down-regulated genes [28], to our list of genes that showed a significant change in regulated in response to sigma-infection. From this comparison, it is clear that there is little overlap between the transcriptional response to bacteria and fungal infections and that of the sigma virus (Table 2). Of the genes that did overlap between the two studies, seven genes were up-regulated in both cases (Table 2). These include the antimicrobial peptide *Metchnikowin*, a translational regulator that is important in immune defense (*Thor*) and five other genes (CG13323, CG10912, CG16743, CG9928, CG15293). Seventeen genes were down-regulated in both studies including four Jonah proteases (*Jonah 25Bii, Jonah 25Biii, Jonah 65Ai* and *Jonah 25Bi*), two related calcium binding proteins that play an important role in many cellular processes (*regucalcin* and *Smp-30*) and 11 other genes (*fit*, CG18594, CG18179, CG12813, CG9090, CG4019, CG13947, CG7322, CG9672, CG9914 and CG3699).

**Table 2:** The effect of the sigma virus compared to bacterial and fungal infection on gene expression.

Sigma virus infected flies		Bacteria and fungus infected males		
Sex	Change expression	Up-regulated^a^	Down-regulated^a^	No change
Female	Up-regulated^b^	2	0	38
	Down-regulated^b^	4	7	67
	No change	77	69	3264
Male	Up-regulated^b^	5	5	230
	Down-regulated^b^	13	12	277
	No change	89	81	3743
Combined	Up-regulated^b^	7	5	266
	Down-regulated^b^	14	17	319
	No change	93	80	4447

Only genes included in both datasets are shown. There is no significant association between the two datasets in any of the three comparisons (Fisher Exact tests on 2×2 contingency tables of genes showing a significant change in expression).

a In the list of 400 ‘Drosophila Immune Related Genes’ identified by De Gregorio [28].

b Significant at *P*<0.02.

**Figure 3:**
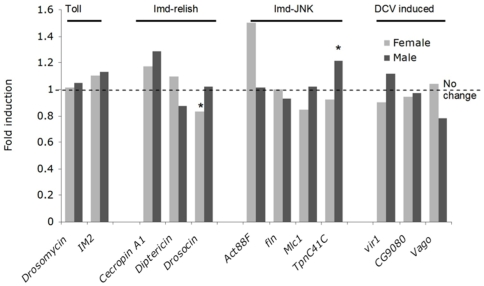
The change in gene expression of genes controlled by known immune pathways in sigma virus infected flies relative to controls. Genes showing a significant change (0.02<*P*<0.05) are labeled*.

Next, we investigated whether sigma activates specific immune signaling pathways (Figure 3). Initially, we investigated how the expression of immunity genes that are known to be induced by either the Toll or IMD pathway changes in response to sigma infection. These genes were also selected to overlap with Figure 1 of Dostert *et al.* [11], who performed a similar analysis to this study, but investigated changes in gene expression in response to DCV-infection. The genes we selected fall into three groups—genes involved in the Toll pathway, the IMD pathway and the Jnk pathway. We looked at the regulation of *IM2* and *Drosomycin* that are up-regulated by the Toll pathway [29], [30], and three antimicrobial peptides *- Cecropin A1*, *Diptericin* and *Drosocin* – that are up-regulated by the transcription factor *Relish* within the IMD pathway [29]. It should be noted that some of these antimicrobial peptides might also be under the control of the Toll pathway [31]. Finally, we looked at *Act88F*, *fln*, *Mlc1* and *TpnC41C*, which are up-regulated by the Jnk pathway [29]. It is clear from Figure 3 that there is no evidence that any of these groups of genes have been up-regulated in response to sigma-infection. In a similar analysis, we compared our data to lists of genes under the control of the Toll and IMD-Relish pathways that were identified by De Gregorio *et al.* [28], and again there is no evidence that these pathways are activated (Table 3). We confirmed that there was no significant difference in the expression of *Attacin A, Drosocin* or *Act88F* by qPCR (Table 4).

**Table 3:** The effect of the sigma virus on the expression of genes controlled by the Toll and IMD pathways (identified by [31]).

Sigma virus infected flies^a^	Bacteria and fungus infected males							
	Up-regulated				Down-regulated			
	IMD	Toll	IMD+Toll	Neither	IMD	Toll	IMD+Toll	Neither
Up-regulated^b^	0	0	0	1	0	0	0	0
Down-regulated^b^	0	0	3	1	2	2	1	3
No change	7	15	13	3	3	11	2	8

a Male and female data combined, similar results were obtained using only male data.

b Significant at *P*<0.02.

**Table 4:** Results of qPCR experiments.

Gene	Male			Female		
	Infected^a^	Uninfected^a^	P^b^	Infected^a^	Uninfected^a^	P^b^
*Attacin-A*	5.51 (1.50)	5.45 (1.49)	0.949	5.26 (1.73)	4.52 (1.07)	0.088
*Drosocin*	14.05 (1.60)	13.65 (2.11)	0.848	14.20 (1.96)	13.05 (0.93)	0.180
*PGRP-SC2*	4.19 (0.45)	4.08 (0.56)	0.949	4.40 (0.33)	3.79 (0.42)	0.011
*Tudor-SN*	5.99 (0.78)	6.18 (0.80)	0.482	7.06 (1.03)	7.61 (0.54)	0.338
*Act88F*	7.82 (0.86)	7.86 (0.83)	0.610	10.46 (1.25)	10.16 (1.04)	0.522

a Shown are the mean ΔCt values (standard deviation) relative to the control ribosomal protein gene, *RpL32*, for seven biological replicates of each gene/treatment.

b Two-tailed *P*-value from Mann-Whitney test.

There has only been one published study on the transcriptional response of Drosophila to sigma virus infection, which used qPCR to measure the expression of a selection of immunity genes [20]. This study found that four antimicrobial peptides and two PGRPs were up-regulated in infected flies. In our study none of these genes were significantly up-regulated in infected flies, despite all six being detected as expressed in both males and females. For two of the genes (*Drosocin* and *Attacin A*), we confirmed this result using qPCR on both the male and female samples (Table 4). The difference between our results and those of Tsai *et al.* [20] is not due to our flies having lower viral titers, as when we measured the copy number of sigma virus genomic RNA relative to *Act88F* by qPCR, we found that our flies had higher viral titers than in this previous study (males: 3.62±1.20; females: 8.14±1.32; average ratio in Tsai *et al.*: 2.3±0.76).

Finally, we compared our data with a microarray analysis of DCV-infected flies (Dostert *et al.*, 2005[32]; Jean-Luc Imler, personal communication). Few genes were up-regulated by both DCV and the sigma virus—of the 85 genes that were significantly up-regulated in response to DCV infection, only nine were up-regulated in response to the sigma virus, and seven were instead down-regulated in sigma-infected flies. There was a greater overlap in the genes that were down-regulated in both DCV and sigma-infected flies—of the 200 genes that were significantly down-regulated in response to DCV infection, 30 were down-regulated in response to the sigma virus, and 11 were up-regulated in sigma-infected flies. Overall, the association between the two studies is marginally non-significant (Fisher Exact Test on 2×2 contingency table of differentially expressed genes: *P* = 0.06).

There were however a few notable immune-related genes that were differentially expressed. The genes *PGRP-SC2*, which is important in dampening the immune response [33], was one of the few genes to be down-regulated in both our male and female datasets (females: ratio infected/uninfected = 0.67, *P* = 0.005; males: ratio = 0.81, *P* = 0.015). We replicated this result using qPCR (females: ratio = 0.66, *P* = 0.011; males: ratio = 0.93, *P* = 0.95). Similarly, *Tudor-SN*, which is involved in RNAi, was down regulated in females, but this was not repeatable using qPCR (Table 4).

### Up- and down-regulated genes

To investigate which biological processes are affected by sigma virus infection, we identified gene ontology (GO) terms that were overrepresented among our up- and down-regulated genes (Table 5). A selection of the genes with these GO terms is listed in Table 6. Among the genes that were down-regulated in sigma-infected males, ribosomal proteins were overrepresented (29 of the 93 genes in one of the two ribosomal subunits). Virtually all of the other GO categories overrepresented among the genes down-regulated in infected males are related to mitochondria. In sigma-infected females, serine proteases were overrepresented among the down-regulated genes, including seven genes from the chymotrypsin superfamily, and six from the Jonah family (note that these categories overlap in the GO annotations). Six chymotrypsin genes were also down-regulated in infected males, including three of the same genes as were detected in infected females. Among the genes that were up-regulated in infected males, genes related to DNA binding and regulating transcription were overrepresented. While in infected females, chorion structural proteins are overrepresented, with three of the nine genes in the genome being significantly up-regulated. Furthermore, three additional chorion proteins (*CP18*, *CP19* and *CP36*) showed up-regulation with *P*-values very close to our detection threshold (*P* = 0.023, *P* = 0.024 and *P* = 0.039 respectively).

**Table 5:** Overrepresented GO terms.

*P*-value	Genes in Genome	Differentially expressed	Domain^a^	GO Term^b^
(a) Down-regulated in females (88 genes)				
4.1E-10	184	13	MF	serine-type endopeptidase activity
1.1E-10	22	7	MF	chymotrypsin activity
(b) Up-regulated in females (46 genes)				
2.6E-06	9	3	MF	structural constituent of chorion
(c) Down-regulated in males (336 genes)				
6.5E-11	377	35	BP	translation
2.0E-11	773	54	BP	cellular biosynthetic process
5.9E-11	337	33	BP	electron transport
1.6E-06	130	15	BP	monocarboxylic acid metabolic process
1.3E-06	15	6	BP	pyruvate metabolic process
2.2E-15	74	20	BP	oxidation reduction
1.6E-10	941	59	BP	biosynthetic process
1.8E-06	489	32	BP	phosphorylation
1.6E-11	458	40	BP	generation of precursor metabolites and energy
2.2E-15	74	20	BP	respiratory electron transport chain
3.2E-14	155	26	BP	oxidative phosphorylation
9.2E-16	71	20	BP	organelle ATP synthesis coupled electron transport
5.6E-09	41	11	BP	mitochondrial electron transport
1.9E-12	333	35	CC	ribonucleoprotein complex
2.9E-17	165	30	CC	ribosomal subunit
3.7E-12	53	15	CC	cytosolic large ribosomal subunit
7.3E-13	40	14	CC	cytosolic small ribosomal subunit
8.3E-18	224	35	CC	cytosol
2.5E-14	128	24	CC	lipid particle
5.7E-17	491	50	CC	mitochondrion
2.1E-13	400	40	CC	organelle membrane
5.4E-16	370	42	CC	mitochondrial part
2.1E-14	223	31	CC	mitochondrial envelope
6.1E-14	187	28	CC	mitochondrial inner membrane
1.1E-15	81	21	CC	mitochondrial respiratory chain
2.8E-16	192	31	MF	structural constituent of ribosome
3.7E-14	705	56	MF	oxidoreductase activity
1.3E-08	235	24	MF	electron carrier activity
1.3E-08	44	11	MF	NADH dehydrogenase activity
(d) Up-regulated in males (293 genes)				
8.47E-06	239	18	MF	sequence-specific DNA binding
2.97E-07	395	27	MF	transcription factor activity
5.28E-08	938	48	BP	regulation of gene expression
7.48E-06	790	38	BP	regulation of transcription, DNA-dependent
8.84E-07	998	47	BP	organ development
1.56E-06	861	42	BP	cell development
1.19E-06	128	14	CC	lipid particle

a BP: biological process; CC: cellular compartment; MF: molecular function.

b Only GO categories overrepresented with *P*<10^−6^ are shown. GO categories that contain over 1000 genes in the genome and categories that were wholly or largely redundant with another category are not shown.

**Table 6:** Significantly up- and down-regulated genes with selected functions.

Males						Females		
Up-regulated		Down-regulated				Up-regulated	Down-regulated	
Serine endopeptidases								
CG11037		Serine protease 6				-	Serine protease 6	
Starving		yippee interacting protein 7 a					yippee interacting protein 7 a	
TER94		epsilonTrypsin		CG17571			Jonah 25Bii a	CG10472^a^
Tripeptidyl-peptidase II		Jonah 25Bi		CG18179 a			Jonah 25Biii	CG11911 a
		CG10472 a		CG3088			Jonah 65Ai	CG1304
		CG1304		CG7542 a			Jonah 65Aiii	CG18179 a
		CG16996 a		CG9672			Jonah 74E a	CG3088
		CG16997 a		CG9673			Jonah 99Ci	CG7829
								CG8329 a
Ribosomal proteins								
Ribosomal proteins:		string of pearls				-	Ribosomal proteins:	
L29		stubarista					L10Ab	
L3		CG6764					L13A	
		overgrown hematopoietic organs 23B					L18A	
		Ribosomal proteins:					LP1	
		L12	L30	S10b	S19a			
		L13	L34b	S11	S25			
		L13A	L7	S14a	S5a			
		L14	L8	S14b	S8			
		L19	LP0	S15Aa	S9			
		L23	LP1	S16				
		L23A	LP2	S18				
Transcription factors								
Hairy/E(spl)-related with YRPW motif		CG11876				SoxNeuro	-	
optomotor-blind-related-gene-1		Deformed				rotund		
Zn finger homeodomain 1		CG33097						
doublesex-Mab related 99B		bicaudal						
Ecdysone-induced prot 74EF								
ftz transcription factor 1								
luna	CG4136							
pannier	bunched							
rotund	midline							
homeobrain	bric a brac 1							
CG15455	abdominal A							
labial	caupolican							
Mnf	POU domain prot 2							
squeeze	forkhead domain 3F							
scribbler	ventral veins lacking							
Chorion Proteins								
-		-				Chorion prot 38	-	
						Chorion prot 15		
						Chorion prot 16		

a chymotrypsin family.

## Discussion

### Immune pathway activation

When Drosophila is infected by bacteria or fungi, the Toll and IMD pathways are activated, leading to the up-regulation of large numbers of genes. These genes include immune effectors such as antimicrobial peptides that are secreted into the hemolymph and defend the flies against the invading pathogens. It is currently unclear whether there is a comparable induced immune defense against viruses. We found that neither genes up-regulated by bacterial or fungal infection, nor the subset of these controlled by the Toll and IMD pathways, are induced in sigma-infected flies. As these pathways control the majority of the genes up-regulated by fungal and bacteria infections [31], any induced immune response to the sigma virus must be controlled by distinct regulatory mechanisms. And although it has been reported that DXV activates the Toll pathway [13] and sigma-virus activates the IMD pathway [20], our results are more similar to with microarray analyses of DCV that found no evidence for the Toll or IMD pathways being activated [11].

An additional pathway implicated in viral infection in Drosophila is the Jak-STAT pathway—genes within the Jak-STAT pathway have been shown to be up-regulated in response to DCV infection, and flies deficient in this pathway are more susceptible to DCV. However, we found that there is little overlap between the genes induced by DCV and the sigma virus, suggesting that the Jak-STAT pathway is not activated by sigma virus. Therefore, we conclude that it is unlikely that Drosophila mounts a general immune response to all viruses.

Why is there little overlap between the genes induced by sigma virus and DCV? One possibility is that DCV, unlike the sigma virus, causes cells lysis and it is this rupturing of cells, and the ensuing tissue damage, that induces an immune response [3]. This hypothesis is consistent with the observation that when flies are fed DCV that results in a relatively benign infection [4] and far fewer genes are induced compared to when flies are injected with the virus [11], [12]. Alternatively, the sigma virus may avoid recognition by the immune system for other reasons. As the sigma virus is only transmitted vertically, it must establish a persistent infection and therefore it must avoid being cleared by the immune response. Avoiding inducing an immune response may be essential for persistent vertically transmitted infections, as the vertically transmitted bacteria *Spiroplasma poulsonii* and *Wolbachia* do not induce an immune response in *D. melanogaster* either [34], [35]. Vertically transmitted infections will also be selected to minimize the harm that the cause to the host, as they rely on their host surviving and reproducing to be transmitted. This may also select for viruses that do not induce a costly transcriptional response in their host.

A previous study by Tsai *et al.* [20] found that four antimicrobial peptides and two PGRPs were strongly induced by the sigma virus. Despite our flies having a higher viral titer, none of these genes were induced in our study. Therefore, the apparent immune response observed by Tsai *et al.* is not always induce by sigma virus infection. The reasons why the results of the two studies differ are unclear. It is possible that only certain fly or viral genotypes induce an immune response, and it is known that fly lines differ greatly in their resistance to the sigma virus [19], at it is not known how the flies in the two studies differ in their resistance genes. Alternatively, the changes in gene expression observed by Tsai *et al.* may not be directly caused by viral infection (for example the sigma infected flies may be more prone to secondary bacterial infections).

### Up- and down-regulated genes

What genes changed in expression in sigma infected flies? In males, the down-regulated genes were strongly enriched for proteins involved in translation, especially ribosomal proteins. Viruses rely on the host's translational machinery to produce proteins, and many viruses—including related virus vesicular stomatitis virus (Rhabdoviridae)—both inhibit host translation and cause viral mRNAs to be preferentially translated over host mRNAs [36]. Furthermore, depleting ribosomal proteins inhibits the replication of DCV in Drosophila cells [37]. The down-regulation of genes involved in translation by the sigma virus probably reflects this interaction, but the biological importance is unclear.

We found that serine proteases were down-regulated in infected flies. Serine proteases have a wide range of functions, including key roles in the regulation of the immune system. The Toll and IMD responses to bacteria and fungi induce and repress a number of serine proteases, some of which have important roles in immune regulation [31], [38]. In females there was an overrepresentation of serine proteases that were down-regulated. Of particular note was an excess of chymotrypsin superfamily serine protease, and several Jonah serine proteases. Several of these genes were also down-regulated in sigma-infected males, making these genes interesting candidates for controlling the fly's response to viruses.

We found that in infected females, genes encoding for chorion proteins were up-regulated. During oogenesis, the chromosomal copies of these genes are amplified up to 10 times, allowing high levels of gene expression [39]. The sigma virus is transmitted through the fly's eggs and it is possible that this relates to the up-regulation of these genes.

### Sex differences in gene expression

Sigma virus infection appears to alter the expression of many more genes in males than in females. For example, with an FDR of 10% we detect over 10 times as many significant genes in males as in females (Figure 1b). Several factors could contribute to this difference. First, the two experiments differ slightly in their replication schemes (10 arrays for males *versus* 8 arrays for females) and statistical power (GEL_50_ = 1.37 for males and 1.52 for females). Thus, we have more power to detect expression differences in the male experiment. This alone, however, cannot explain the large discrepancy in the number of differentially expressed genes. If we exclude two (GEL_50_ = 1.43) or even four (GEL_50_ = 1.57) of the male replicates, we still detect a larger fraction of significant genes in the male experiment (Figure 1b). A second factor could be sex-biased gene expression, as the genes that we detected differ between the two experiments. Using the sex-bias classifications of Gnad and Parsch [40], we find that 59% of the male-biased genes on the array are detected in males, while only 16% are detected in females. In contrast, 43% of female-biased genes on the array are detected in females, while only 28% are detected in males. Sex-biased genes, however, are not over-represented among those whose expression was significantly altered by the sigma virus. Male-biased genes comprise 18% of the genes detected in males, but only 14% of the genes significantly affected by sigma infection. Similarly, female-biased genes comprise 18% of the genes detected in females, but only 11% of the genes significantly affected by the sigma infection.

It is also possible that the difference between males and females results from intersexual differences in the mechanism of viral transmission or host defense. Sigma virus is transmitted along with sperm and there appear to be specific barriers to it entering the male germline [4]. Furthermore, genes that cause variation in the transmission of the virus often affect transmission through sperm, but not eggs [19]. This may reflect a qualitative difference in the nature of the infection in males, or indicate that specific resistance responses are triggered in the male germline, leading to a greater transcriptional response to infection. Alternatively, males and females may invest differently in their immune defenses [41], and this may be reflected in sex differences in the transcriptional response to sigma infection.

A final possibility is that, in general, male gene expression is more sensitive to genetic and/or environmental perturbations than female gene expression. Some support for this comes from the observation that, among laboratory strains, greater gene expression variation is observed among males than among females [42]. However, more experimental comparisons of male and female transcriptional responses to a common treatment are necessary to determine the generality of this observation.

## Supporting Information

File S1 The relative expression level, 95% confidence limits of expression levels in males and females, and probability of differential expression of each gene between infected and uninfected males
(1.28 MB XLS)

File S2 The relative expression level, 95% confidence limits of expression levels in males and females, and probability of differential expression of each gene between infected and uninfected females
(1.00 MB XLS)

File S3 The list of significantly differentially expressed genes (P<0.02) in which the CG numbers have been updated to match v49 of ENSEMBL
(2.20 MB XLS)
